# Scale Development for COVID-19 Vaccine Hesitancy by Integration of Socio-Demographic and Psychological Factors

**DOI:** 10.3390/vaccines11061052

**Published:** 2023-06-01

**Authors:** Eliza Sharma, Subhra Mondal, Subhankar Das, Vasiliki G. Vrana

**Affiliations:** 1 Symbiosis Institute of Business Management Bengaluru, Symbiosis International (Deemed University), Karnataka 560100, India; eliza.sharma@sibm.edu.in; 2 The Honors Programme, Department of Marketing, South Star Management Institute, Duy Tan University, Da Nang 550000, Vietnam; subhraranimondal@duytan.edu.vn; 3 Department of Business Administration, School of Economics and Administration, The Campus of Serres, International Hellenic University, 62124 Serres, Greece

**Keywords:** cognitive fit, emotional fit, psychological factors, sociodemographic elements, COVID-19, vaccine hesitancy, SEM

## Abstract

Vaccination is the most cost-effective way to maintain population health. However, it can only be effective if widespread acceptance is held. The efficacy of COVID-19 vaccines depends on their favor. When countries start to vaccinate their citizens, there is a certain level of skepticism about the effectiveness of the vaccines. The hesitancy study on vaccines has gained momentum following the pandemic. However, few studies have examined the link between the psychological and sociodemographic factors influencing the fit. This paper proposes integrating the concepts of the information systems success and stimulus–organism–response into a cognitive fit theory framework to explore the integration of psychological and sociodemographic factors in the receivers’ reactions (*n* = 1510). This study analyses the factors that influence the hesitancy of vaccines and the public’s refusal in Asia and Europe. Receivers’ reactions were assessed to various stimuli and we explored the link between psychological and sociodemographic elements and the concept of fit. Two surveys were conducted following the scale development of Mackenzie. The first was to develop the fit scale, while the second was to validate the fit scale. The results of the second survey were analyzed using structural equation modelling. The results indicate that the scale’s fit development is valid and reliable. The quality of the vaccine information, the psychological characteristics of the vaccine system, and vaccine receivers’ satisfaction are also beneficial factors for emotional and cognitive fit. Maintaining the vaccines’ quality and efficiency can help improve the fit between sociodemographic and psychological characteristics. It can also enhance receivers’ satisfaction and encourage continued vaccine administration. This study is regarded as one of the first to examine and develop an emotional and cognitive fit scale for practitioners and researchers.

## 1. Introduction

Vaccination can help prevent the spread of the disease, which has caused significant economic and social damage. Since the 18th century, vaccines have been used to avoid illnesses such as smallpox, polio, and measles [1]. Due to the emergence of the COVID-19 pandemic, the need for vaccines has become more urgent. Some people are eager to get vaccinated against the COVID-19 pandemic, while others are hesitant [2].

The exploitation and historical mistreatment of minority groups by the medical community have led to a distrust of institutions and health care workers [3]. This issue has been exacerbated by COVID-19′s disproportionate impact on these communities. It is essential that the public has access to accurate information and trusts medical professionals [4]. Concerns about the safety of the shots also cause vaccine hesitancy. Some individuals hesitate to get vaccinated due to the potential side effects, while others doubt the jab’s effectiveness [5]. The COVID-19 shots have been extensively tested and are safe and effective. Millions of vaccine doses have been distributed to the general population and are safe and effective [6]. Unfortunately, despite the positive safety record of the shots, vaccine hesitancy continues to be a significant issue that needs to be resolved to end the pandemic and achieve herd immunity [7]. As a community, we must work together to stop the spread of the pandemic and safeguard everybody’s well-being [8].

Vaccine hesitancy is a growing public health concern. It refers to the delay in acceptance or refusal of vaccines despite the availability of vaccination services [9]. There are many factors that contribute to vaccine hesitancy, including sociodemographic variables such as age, education level, income, race, and ethnicity [10,11,12,13]. Education level is one of the most significant sociodemographic factors influencing vaccine hesitancy [14]. Studies have shown that individuals with lower levels of education are more likely to be hesitant about vaccines [15]. This may be due to limited access to vaccine information or a lack of understanding of the importance of vaccination [16]. Age is another essential sociodemographic variable that impacts vaccine hesitancy. Older individuals may be less likely to accept vaccines, while younger individuals may be more hesitant due to concerns about vaccine safety and efficacy [17]. Income level also plays a role in vaccine hesitancy, with individuals in lower income brackets being more likely to be hesitant [18]. This may be due to a lack of access to health care or concerns about the cost of vaccines. Race and ethnicity are critical sociodemographic variables in vaccine hesitancy [19]. Some minority groups have expressed distrust in the health care system due to past experiences of discrimination and mistreatment [20]. This may lead to a lack of trust in vaccines and vaccination services. It is important to understand the factors that contribute to vaccine hesitancy in order to develop effective interventions to address it [21]. By doing so, we can help to ensure that everyone has access to the benefits of vaccination.

Psychological factors such as fear, mistrust, and conspiracy beliefs also played a role [22]. Fear of side effects and distrust of the medical establishment were significant predictors of vaccine hesitancy [23]. Conspiracy beliefs about vaccines: the belief that vaccines are part of a government agenda and the belief that they cause autism. Ultimately, the multi-faceted approach of effective communication, building trust, and addressing fears and mistrust paid off [24]. As more and more people were vaccinated, the spread of COVID-19 was ultimately controlled. The world was able to breathe a collective sigh of relief, knowing that the vaccine had helped to put an end to the crisis [25]. Effective communication and trust are essential in addressing vaccine hesitancy. Public health officials and health care providers should provide accurate vaccine information to address fears and mistrust [26]. Moreover, building trust with marginalized communities is crucial in addressing vaccine hesitancy among people of color.

COVID-19 vaccine hesitancy is a complex issue that involves sociodemographic and psychological factors. Here, the authors explored the sociodemographic and psychological factors contributing to COVID-19 vaccine hesitancy. Addressing vaccine hesitancy requires a multi-faceted approach that involves effective communication, building trust, and addressing fears and mistrust [27]. Addressing these factors can increase vaccine uptake and ultimately control the spread of COVID-19. In conclusion, sociodemographic variables play a critical role in vaccine hesitancy. Understanding these variables can help health care professionals and policymakers develop targeted interventions to address vaccine hesitancy and improve vaccine acceptance rates [28].

The COVID-19 pandemic profoundly impacted the world, and vaccination is one of the most important public health measures to mitigate the spread of the virus. However, vaccine hesitancy is a significant challenge, and several factors can influence a person’s decision to get vaccinated. One factor that has been shown to have an impact on COVID-19 vaccine intention is emotional fit. Emotional fit refers to the extent to which a person’s emotional response to a situation is consistent with their values and beliefs. For example, someone highly anxious about COVID-19 is more likely to be motivated to get vaccinated than someone not worried.

Various studies have found that emotional fit is also associated with COVID-19 vaccine intention [29,30]. People who reported feeling anxious about COVID-19 were more likely to intend to get vaccinated than people who did not feel anxious [31]. Another study found that people who reported feeling angry about COVID-19 were more likely to intend to get vaccinated than people who did not feel angry [32]. The relationship between emotional fit and COVID-19 vaccine intention is expected due to several factors. First, emotions can influence people’s attention and information processing [30]. When people feel anxious or angry, they are more likely to pay attention to information about COVID-19 and be motivated to take action to protect themselves [31]. Second, emotions can influence people’s decision making. When anxious or angry, people are more likely to make decisions consistent with their emotional state. In the case of COVID-19, people feeling anxious or angry are more likely to decide to get vaccinated [32]. In addition to emotional fit, several other factors can influence COVID-19 vaccine intention. These factors may include:

- Perceived risk of COVID-19: People who perceive themselves to be at high risk of contracting COVID-19 are more likely to intend to get vaccinated.
- Trust in vaccines: People who trust vaccines are more likely to intend to get vaccinated.
- Perceived benefits of vaccination: People who perceive the benefits of immunization to be greater than the risks are more likely to intend to get vaccinated.
- Perceived barriers to vaccination: People who perceive barriers to immunization, such as cost or lack of access, are less likely to intend to get vaccinated.

Other studies found that cognitive fit is another critical factor for psychological makeup [33,34]. It is a cognitive theory that suggests that people are more likely to understand and remember information presented in a way consistent with their existing knowledge and beliefs [34]. This theory has been applied to various domains, including health behavior. In the context of COVID-19 vaccination, the cognitive fit could influence the intention to get vaccinated in a few ways. For example, people who strongly believe in the importance of vaccination may be more likely to understand and remember information about the COVID-19 vaccine if it is presented in a way that is consistent with their beliefs [35]. Conversely, people with a negative attitude towards vaccination may be more likely to dismiss information about the COVID-19 vaccine if it is presented in a way inconsistent with their existing beliefs [36].

The cognitive fit is a cognitive theory that could influence the intention to vaccinate against COVID-19 in several ways. In addition to affecting understanding and memory, the cognitive fit could also influence the perceived credibility of information about the COVID-19 vaccine. People who perceive the information as credible are more likely to be persuaded. Therefore, if information about the COVID-19 vaccine is consistent with people’s existing knowledge and beliefs, they are more likely to perceive it as credible and be persuaded. By submitting information about the COVID-19 vaccine consistent with people’s existing knowledge and ideas, we can increase their understanding, memory, and perceived credibility of the information, which could lead to increased intention to get vaccinated. It is important to note that these factors are not mutually exclusive. For example, a person anxious about COVID-19 is also more likely to perceive themselves at high risk of contracting the virus. Additionally, a person who trusts vaccines is also more likely to perceive the benefits of vaccination to be greater than the risks.

Vaccine hesitancy is a growing concern worldwide, especially in the current pandemic. The World Health Organization (WHO) has listed vaccine hesitancy as one of the top ten threats to global health [37]. Although vaccines have been proven effective in preventing infectious diseases, vaccine hesitancy has caused a decrease in vaccination rates, leading to the resurgence of some previously controlled diseases [38]. Understanding the factors contributing to vaccine hesitancy is vital in promoting vaccine acceptance and improving public health. Despite the significance of sociodemographic and psychological factors in vaccine hesitancy, there is a research gap in understanding the complex interplay between these factors. Most existing studies on vaccine hesitancy have focused on either sociodemographic or psychological factors, with limited attention to the interaction between these factors. There is a need for more comprehensive and integrated studies that explore the combined influence of sociodemographic and psychological factors on vaccine hesitancy. Understanding the complex interplay between these factors can help identify the most effective interventions to promote vaccine acceptance.

The concept of fit has been studied in various fields, such as marketing, psychology, and management [39]. It can be used to analyze the relationship between an individual’s experiences and their outcomes. Although there is a lot of literature about the fit between people and their environments, studies on the effects of emotional fit and cognitive fit on the development of vaccines are not yet available [40]. For instance, according to the concept of cognitive fit, solving problems involves spatial and symbolic elements. We have examined the literature on the cognitive fit and information system (news) of vaccines in various databases, and there is currently no evidence supporting the link between these conditions and vaccine hesitancy [41]. Due to the lack of integration of psychological and sociodemographic elements into studies, vaccine hesitancy negatively affects the research process [42]. For instance, in our study, the psychological fears of vaccine recipients are influenced by the information system’s news and demographic variabilities [43]. Although there are many features of information technology and vaccine hesitancy, it is only effective if the virtual information it provides can support the psychological makeup of the vaccine recipients [44]. It is also essential to consider the various factors that affect the development and implementation of a virtual news environment, such as its high fidelity and user control—having the necessary cohesion between the virtual and real worlds.

### Rationale of Research

Cognitive and emotional fit is a term used to describe the alignment of an individual’s cognitive and emotional abilities with the demands of a particular role which is the intention towards vaccine after feeling satisfied or dissatisfied (how much the individual is inclined to take the jab or not) [45]. Social and psychological factors, on the other hand, are a broader set of factors that can influence an individual’s ability to perform better towards vaccine intention. These factors include personality, motivation, and social support [46].

Social and psychological factors focus on the individual’s beliefs, attitudes, and experiences that may influence their vaccination decision. It can be affected by various factors, such as personal experiences with vaccines, information from trusted sources, and social norms [47]. It can be addressed through multiple interventions, such as providing accurate information about vaccines, addressing safety concerns, and building social support for vaccination. Cognitive and emotional fit focuses on the individual’s ability to understand and process information about vaccines and their ability to manage the emotional response to vaccination [48]. It can be influenced by cognitive ability, emotional intelligence, and personality factors. It can be addressed through interventions that improve the individual’s ability to understand and process information and help them manage their emotional response to vaccination [49].

It is important to note that social and psychological factors and cognitive-emotional fit can influence COVID-19 vaccine hesitancy. In some cases, one factor may be more important than the other. For example, an individual who has had a negative experience with a vaccine may be more likely to be hesitant to get vaccinated, regardless of their cognitive ability or emotional intelligence [50]. By understanding the social and psychological factors and cognitive-emotional fit that may influence an individual’s decision to get vaccinated, we can develop more effective interventions to increase vaccination rates. Here are some examples of how social and psychological factors and cognitive-emotional fit can influence COVID-19 vaccine hesitancy:

Social and psychological factors

- Personal experiences with vaccines: Individuals with a negative experience with a vaccine, such as a severe allergic reaction, are more likely hesitant to get vaccinated.
- Information from trusted sources: Individuals are more likely to trust information about vaccines from sources they trust, such as their doctor, family, or friends.
- Social norms: Individuals are more likely to get vaccinated if they see their friends, family, and community members getting vaccinated.

Cognitive-emotional fit

- Cognitive ability: Individuals with lower cognitive ability may have difficulty understanding vaccine information.
- Emotional intelligence: Individuals with low emotional intelligence may have difficulty managing their emotional response to vaccination, such as fear or anxiety.
- Personality: Individuals with certain personality traits, such as anxiety or distrust, may be more hesitant to get vaccinated.

By understanding the social and psychological factors and cognitive-emotional fit that may influence an individual’s decision to get vaccinated, we can develop more effective interventions to increase vaccination rates. It is important to note that cognitive, emotional fit and social and psychological factors are not mutually exclusive. They are often interrelated [51,52,53,54]. For example, an individual with high cognitive ability may be more likely to take good decisions successfully that require a lot of problem-solving. However, they may also be more likely to experience stress if they do not have adequate social support [55,56,57,58]. By understanding the cognitive, emotional fit, and social and psychological factors important for vaccine hesitancy, vaccine makers and government agencies can better decide who to hire, place, train, and develop. It can lead to a more productive and satisfied workforce.

This study aims to explore the optimal combination of information technology and vaccine recipients’ psychological and sociodemographic makeup to develop effective strategies for addressing the complex COVID-19 vaccine hesitancy issues. Although the concept of fit has been studied in various contexts, not much is known about the application of this concept to vaccine hesitancy. This study explores the relationship between people’s emotional and cognitive fit regarding vaccine hesitancy. The study uses the S-O-R model to measure recipients’ reactions to varying stimuli and examine the integration of various information sources [59]. Unfortunately, no standard questionnaire can be used to investigate the link between a vaccine’s fit and information integration. This study aims to develop a scale model that will allow us to analyze the integration of information sources in real and virtual environments. There are two primary objectives of this study such as.

1. To develop a scale model that will allow us to analyze the link between users’ cognitive and emotional fit in the context of vaccine hesitancy.
2. To analyze the sociodemographic and psychological factors influencing users’ emotional and cognitive fitness in the vaccine hesitancy context.

In this paper, we discuss the cognitive fit of vaccines to the real-world context of their recipients. We also explore the various phenomena that influence this concept. We aim to develop a new theoretical framework for analyzing virtual and real-world information integration in vaccine uptake. Cognitive fit refers to assessing the appropriateness of information related to a vaccine for the recipient’s context. The more accurate the information is, the more likely the recipient will experience the unity of real and virtual communication. Vaccine recipients can feel safe if the information they receive meets their needs. Vaccination hesitancy can be minimized by validating the facts and exposing the recipient to more accurate information. The emotional fit of vaccines is related to the availability of various types and their side effects. It helps the recipient feel safe and secure while receiving the immunization.

## 2. Literature Review

### 2.1. The Success Model for Information Systems

The quality of information is a measure of the output of an information system. It involves various factors such as completeness, accuracy, timeliness, mobility, and solvability. The success factors include the quality of the system, the information quality, the organization’s impact, the individual’s satisfaction, service quality, an expanded use section, and a conclusion that considers the net benefits of the system [60]. Information quality is a vital factor that can be regarded when it comes to assessing the effectiveness of an information system model. For social media platforms, information quality can be affected by the measurement method [61]. For instance, people may not trust the information a stranger post on a website.

An information system’s effectiveness is evaluated based on various factors such as ease of use, accessibility, and reliability. System quality is an essential factor when it comes to assessing the effectiveness of a social media information system. It also involves factors such as response time and system integration [62]. The characteristics of an information system can help improve its efficiency, reduce the likelihood of vaccine hesitancy, and facilitate information usage. On the other hand, having a reliable update information system helps developers ensure their data are reliable.

### 2.2. The Intention and Satisfaction of Information Users

The satisfaction index is a measure of how users feel about a product or service after they have used it [63]. Although different perspectives and definitions of satisfaction exist, it is generally regarded as a combination of negative and positive feelings or attitudes, and it can be triggered by various factors, such as the information they receive or how it affects them [64,65,66]. For instance, if users are dissatisfied with the information they receive, they might search for something new. Users’ satisfaction after using a specific information system can influence their intention to continue using it. It is because it can predict their future usage. For instance, if users are delighted with their current information system, they are more likely to continue consuming it [67]. So, if good communication brings out trust and well-being, vaccine takers will rely on and continue to believe the source of information.

### 2.3. Cognitive Fit and Emotional Fit for Vaccine Information Use

Cognitive fit is essential when processing and acting on vaccine information. Considering how the data align with our prior beliefs, values, and attitudes is critical. Let us strive to approach vaccine information with an open mind and a willingness to engage with different perspectives. In recent times, vaccine information use has become increasingly important. With the COVID-19 pandemic, there has been an immense surge in the number of people accessing vaccine-related information. However, not all individuals process this information in the same way. Cognitive fit, or the degree of match between an individual’s cognitive processes and the information they are processing, plays a crucial role in the effectiveness of vaccine information use [68]. Cognitive fit is determined by several factors, including an individual’s cognitive style, prior knowledge, and personal beliefs. Individuals with a high cognitive fit are likelier to understand and make decisions based on the information presented.

On the other hand, individuals with low cognitive fit may struggle to understand the information and make poor decisions. It is essential to consider the cognitive fit of the data [69]. It can be achieved by presenting information in a way that is tailored to individuals’ cognitive styles and beliefs. For example, visual aids such as infographics and videos may be more effective for individuals with a visual cognitive style. Similarly, narrative information may be more effective for individuals who prefer storytelling.

In conclusion, the cognitive fit is crucial in vaccine information use [70]. By considering an individual’s cognitive style, prior knowledge, and personal beliefs, we can present information in a way that is more effective and leads to better decision making. It can ultimately result in increased vaccine uptake and improved public health outcomes.

As the COVID-19 pandemic continues to ravage the world, the importance of vaccines in controlling the spread of the virus cannot be overstated. However, vaccine hesitancy, or the reluctance or refusal to vaccinate, remains a significant barrier to achieving herd immunity [71]. One factor that has been found to influence vaccine hesitancy is emotional fit, or the degree of alignment between a person’s emotions and the message being conveyed. Emotional fit is particularly relevant in the context of vaccine information use. A study published in the Journal of Health Communication found that emotional fit significantly predicted vaccine information-seeking behavior [72]. Specifically, individuals who experienced negative emotions towards vaccines were less likely to seek out information about vaccines, while those who experienced positive emotions were more likely to seek out information. The study’s authors suggest that emotional fit may be related to the perceived relevance of vaccine information. When individuals feel emotionally aligned with a message, they are more likely to perceive it as relevant to their lives and, thus, more likely to seek additional information.

On the other hand, when individuals feel emotionally misaligned with a message, they are less likely to perceive the message as relevant and less likely to engage with it. So, what can be done to increase emotional fit and promote vaccine information use? One strategy is to tailor vaccine messages to the emotional needs of the target audience. For example, notes that emphasize the importance of protecting loved ones may be more emotionally salient to some individuals than messages that emphasize the scientific benefits of vaccination. Additionally, it may be helpful to acknowledge and address common emotional concerns around vaccines, such as fears about side effects or mistrust of the medical establishment.

In conclusion, emotional fit is vital in vaccine information use and hesitancy. By tailoring vaccine messages to the emotional needs of the target audience and addressing common emotional concerns, we may be able to increase emotional fit and promote vaccine uptake.

### 2.4. Stimulus–Organism–Response and Vaccine Information Processing by Users

Stimulus–organism–response (SOR) theory is a psychological theory that describes how people respond to stimuli in their environment. SOR theory suggests that a catalyst, such as information about a vaccine, will be processed by an organism, such as a person, and will result in a response, such as a decision to get vaccinated or not [73]. Regarding vaccine information processing, many factors can influence a person’s response. One of the most important factors is the source of the information. People are more likely to trust information about vaccines from a reliable source, such as a health care professional than from an unreliable source, such as social media [74]. Another critical factor is the content of the information. People are more likely to respond positively to vaccine information that is clear, concise, and easy to understand. They are also more likely to react positively to reports highlighting the benefits of vaccination and addressing any concerns or questions they may have [75]. Finally, how the information is presented can influence a person’s response. For example, data presented in a visual format, such as an infographic or video, may be more effective at conveying information than in a text format. For promoting vaccine uptake, it is essential to consider the following factors when designing a vaccine information campaign; by ensuring that information is reliable, transparent, and presented effectively, we can help promote informed decision making and increase vaccine uptake.

### 2.5. Demographic Factors and Vaccine Information Processing

Demographic factors such as age, gender, income, and education are crucial in determining vaccine hesitancy and vaccine information use [76]. Several studies have shown that younger people are more likely to be hesitant about vaccines and less likely to use vaccine information. Similarly, people with lower education and income levels are also more likely to be uncertain about vaccines [77]. One reason for this hesitancy is misinformation and a lack of trust in the health care system. Social media and other online platforms have made spreading false information easier, leading to confusion and distrust in vaccines. In addition, some people may feel that they have not been given enough information to make an informed decision about vaccines. It is important to understand the reasons behind it and develop targeted interventions to address those reasons to counter vaccine hesitancy [36,77]. Health care providers can be crucial in providing accurate information and building trust with patients. They can also tailor their messaging to specific demographic groups to address their concerns and increase vaccine uptake. In addition, public health campaigns can effectively increase vaccine information use and reduce hesitancy [78]. These campaigns can use a variety of channels, such as social media, television, and print media, to reach different demographic groups. Overall, understanding the role of demographic factors in vaccine hesitancy and vaccine information use is crucial for developing effective interventions and increasing vaccine uptake. By addressing the concerns and needs of different groups, we can work towards building a more equitable and healthy society.

### 2.6. Social Factors and Vaccine Information Processing

Vaccines are crucial in preventing the spread of infectious diseases, but vaccine hesitancy remains a significant barrier to achieving herd immunity [79]. One factor contributing to vaccine hesitancy is how people process information about vaccines. Social factors such as trust in medical professionals, political beliefs, and cultural norms can all influence how people interpret vaccine information [80,81,82]. Confidence in Medical Professionals is one of the most significant social factors influencing vaccine information processing is trust in medical professionals [83,84,85]. People who have a high level of trust in their health care providers are more likely to see vaccines as safe and effective [86].

Political beliefs are another social factor affecting vaccine information processing is political beliefs. It can lead to vaccine hesitancy and a preference for alternative therapies. Vaccine hesitancy is often associated with certain political ideologies, such as libertarianism or conservatism. People with these beliefs may be more skeptical of government recommendations and more likely to believe in conspiracy theories or misinformation about vaccines [87]. Conversely, people with low trust in medical professionals may be more likely to believe misinformation about vaccines and be hesitant about getting vaccinated. Cultural norms can also play a role in vaccine information processing. In some cultures, there may be a strong emphasis on natural remedies and a distrust of Western medicine.

Additionally, some cultural or religious beliefs may conflict with vaccine recommendations, making it more difficult for individuals to make informed decisions about vaccination. Vaccine information processing is a complex and multi-faceted issue that is influenced by a range of social factors. To effectively address vaccine hesitancy, it is crucial to understand and address these social factors [88]. Health care providers and public health officials can play a role in building trust and addressing misinformation while acknowledging and respecting cultural beliefs and values [89,90].

**Figure 1:**
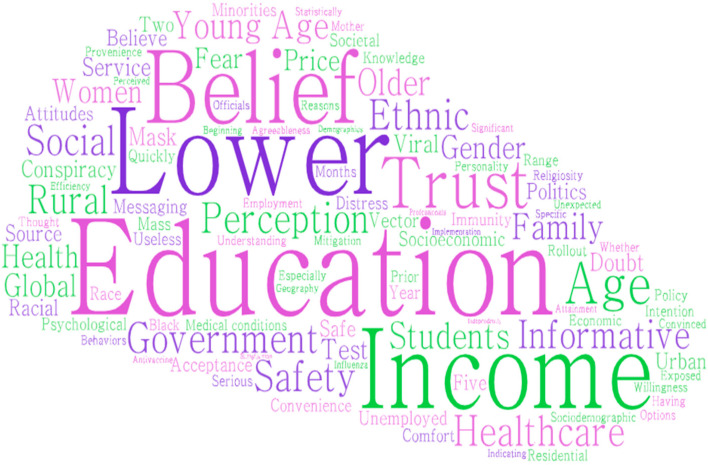
Word Cloud for Factors from Vaccine Hesitancy Literature. Source: Authors’ analysis.

From recent studies of vaccine hesitancy for COVID-19, we came across various prominent factors apart from our considerations for this study [51,52,53,54,55,56,57,58,80,81,82,83,84,85]. Researchers have overviewed 19 published literature from 2020 to 2022 and for holistic integration for the already studied factors, researchers here plotted a word cloud which is presented in this Figure 1.

## 3. Hypothesis Development for Different Constructs

### 3.1. Relation between Information Quality with the Cognitive and Emotional Fit for Vaccine Hesitancy

- Information quality refers to the accuracy, completeness, and reliability of the available information. Regarding vaccines, accurate and reliable information is essential to help people make informed decisions about vaccination [59,60,61].
- Cognitive fit matches how information is presented and how our brains process information. When data are presented in a way that matches our cognitive processes, we are more likely to understand and remember it [64,65,66].

Regarding vaccine hesitancy, it is essential to consider both concepts. People hesitant about vaccines may receive inaccurate or incomplete information, leading to misunderstandings and misinformation. Additionally, how information about vaccines is presented may not be aligned with the cognitive processes of hesitant people, making it difficult for them to understand and remember the information. Ensuring that accurate and reliable information is available to address vaccine hesitancy is essential. This information should be presented in a way that is aligned with the cognitive processes of hesitant people, making it easier for them to understand and remember the information.

In conclusion, information quality and cognitive fit are essential when addressing vaccine hesitancy. By ensuring that accurate and reliable information is available and presented to match the cognitive and emotional processes of hesitant people, we can help address vaccine hesitancy and promote public health. So, the hypotheses are as follows:

**H_1_**: Information quality strongly and significantly affects cognitive fit for vaccine hesitancy of recipients.

**H_2_**: Information quality strongly and significantly affects emotional fit for vaccine hesitancy of recipients.

### 3.2. Relation between Health Care System Quality with the Cognitive and Emotional Fit for Vaccine Hesitancy

The quality of a health care system can have a significant impact on vaccine hesitancy. When individuals do not feel that the health care system understands their unique needs and concerns, they may be less likely to trust the system’s recommendations for vaccination [67,68,69]. This lack of trust can lead to vaccine hesitancy and serious public health consequences. There are several things that health care systems can do to address vaccine hesitancy. One important step is to prioritize the development of patient-centered care that focuses on meeting the unique needs and concerns of everyone. This can be achieved using patient-centered communication strategies, such as motivational interviewing and shared decision making. In addition, health care systems must work to build trust with their patients by providing transparent and accurate information about vaccines and their safety and efficacy [69,70,71]. This can be achieved using educational materials, social media campaigns, and community outreach efforts. Ultimately, improving the quality of health care systems is essential for addressing vaccine hesitancy and ensuring that all individuals have access to the vaccines they need to protect their health and the health of their communities. So, the hypotheses are:

**H_3_**: Health care system quality strongly and significantly affects cognitive fit for vaccine hesitancy of recipients.

**H_4_**: Health care system quality strongly and significantly affects the emotional fit for vaccine hesitancy of recipients.

### 3.3. Relation between Demographic Factors with the Cognitive and Emotional Fit for Vaccine Hesitancy

Vaccine hesitancy is a complex issue that can have far-reaching consequences for public health. Understanding the underlying factors contributing to this hesitancy can help public health officials and researchers address it better.

There are several factors that contribute to vaccine hesitancy, including:

- Demographic factors: Age, education level, income, and race are all linked to vaccine hesitancy. For example, younger individuals and those with lower education and income levels are more likely to be hesitant about vaccines. Additionally, individuals from certain racial and ethnic groups may be more uncertain due to historical and current issues of mistrust in the medical community [67,68,69,70].
- Cognitive and emotional factors: Individuals with higher anxiety or fear may be more hesitant to get vaccinated. At the same time, those with a stronger sense of community responsibility may be more likely to get vaccinated [60,61,62]. Additionally, individuals with certain beliefs or values may be hesitant due to concerns about safety or efficacy.

Understanding the relationship between demographic factors and cognitive and emotional fit for vaccine hesitancy is critical in effectively addressing this issue. Public health officials and researchers must take a multi-faceted approach that considers individual and societal factors contributing to vaccine hesitancy. By doing so, we can work towards increasing vaccine uptake and protecting public health. So, the hypotheses are:

**H_5_**: Demographic factors strongly and significantly affect cognitive fit for vaccine hesitancy of recipients.

**H_6_**: Demographic factors strongly and significantly affect recipients’ emotional fit for vaccine hesitancy.

### 3.4. Relation between Sociological Factors with the Cognitive and Emotional Fit for Vaccine Hesitancy

Vaccine hesitancy is a complex issue that can have far-reaching consequences for public health. There are several factors that contribute to vaccine hesitancy, including:

- Distrust in the medical community: Distrust can be rooted in historical experiences, such as unethical medical experimentation on marginalized communities. Additionally, misinformation and propaganda can perpetuate mistrust in the medical community, leading to vaccine hesitancy [59,60,61,62,63].
- Social norms and cultural beliefs: For instance, some communities may reject vaccination because it is against their cultural or religious beliefs. Additionally, social norms can influence people’s attitudes towards vaccination. For instance, if a person’s social circle is against vaccination, they may adopt a similar stance [79,80].
- Cognitive and emotional fear: Cognitive fear refers to the fear of vaccine side effects or adverse reactions, while emotional fear pertains to the fear of needles, pain, or anxiety. These fears are valid, and it is essential to acknowledge them. However, it is equally vital to debunk myths and misinformation surrounding vaccination and provide accurate information.

To address vaccine hesitancy, it is essential to understand the sociological factors contributing to it. Medical professionals and policymakers must work to build trust in the medical community, provide accurate information about vaccines, and address cultural and social norms that perpetuate vaccine hesitancy. So, here, the authors conclude that:

**H_7_**: Sociological factors strongly and significantly affect cognitive fit for vaccine hesitancy of recipients.

**H_8_**: Sociological factors strongly and significantly affect recipients’ emotional fit for vaccine hesitancy.

### 3.5. Relation between Cognitive Fit and Vaccination Receiver Satisfaction for Vaccine Hesitancy

Vaccine hesitancy has been a significant concern for public health officials worldwide, particularly during the COVID-19 pandemic [70]. One factor that has been found to impact vaccine hesitancy is cognitive fit, or the degree to which the information presented to a person matches their cognitive processes. Research has shown that when information is presented in a way that matches a person’s cognitive processes, they are more likely to understand and retain the data [73]. It, in turn, can lead to greater satisfaction with the decision to receive a vaccine. So, here, the authors conclude that:

**H_9_**: Cognitive fit strongly and significantly affects vaccination receiver satisfaction for vaccine hesitancy.

### 3.6. Relation between Emotional Fit and Vaccination Receiver Satisfaction for Vaccine Hesitancy

- Emotional fit is the alignment between an individual’s emotional state and communicated message [74].
- Vaccination receiver satisfaction is the level of satisfaction an individual has with the vaccination experience [66].
- Vaccine hesitancy is the reluctance or refusal to vaccinate oneself or one’s children [68].

The text discusses the importance of emotional fit in both vaccination receiver satisfaction and vaccine hesitancy. When the emotional state of the receiver and the message being communicated are not in alignment, it can lead to confusion and mistrust. This is especially important in the context of vaccination, as trust in medical professionals and government agencies is a crucial factor in vaccine acceptance [69,70,71,72,73] Studies have shown that emotional fit is essential for vaccination receiver satisfaction.

Some studies found that individuals who received a flu vaccine were more satisfied with the vaccination experience when the health care provider demonstrated empathy and understanding towards their concerns. Similarly, another study found that parents were more likely to vaccinate their children when health care providers took the time to address their concerns and showed empathy towards their decision-making process [74,75,76].

Understanding the relationship between emotional fit and vaccine hesitancy can also help develop effective interventions to promote vaccine acceptance. For example, health care providers can take the time to address the concerns of vaccine-hesitant individuals and provide them with accurate information about the benefits and risks of vaccination. Additionally, public health campaigns can be designed to promote emotional fit by using messaging that aligns with the emotional states of the target audience. In conclusion, emotional fit is a crucial factor in both vaccination receiver satisfaction and vaccine hesitancy. By understanding this relationship, health care providers and public health officials can develop effective strategies to promote vaccine acceptance and improve public health outcomes. So, here, the authors conclude that:

**H_10_**: Emotional fit strongly affects vaccination receiver satisfaction for vaccine hesitancy.

### 3.7. Relationship between Vaccination Receiver Satisfaction and Intention to Receive Future Vaccination Doses for Vaccine Hesitancy

One factor that can contribute to vaccine hesitancy is satisfaction with a previous vaccination experience. Individuals who are satisfied with their previous vaccination experience are more likely to intend to receive future vaccination doses. There are several things that health care providers can do to improve the vaccination experience and increase vaccine uptake. These include:

- Providing clear information about the benefits and risks of vaccination.
- Addressing concerns and questions.
- Minimizing pain or discomfort during the vaccination process.

By promoting pride in the vaccination experience, public health officials and health care providers can help address vaccine hesitancy and promote vaccination uptake [74,75,76].

Here are some additional details from the text that support these conclusions:

- Individuals who reported high levels of satisfaction with a flu vaccine were more likely to intend to receive future flu vaccines [36].
- Satisfaction with a previous vaccination was associated with receiving the next dose [78].
- Health care providers can focus on improving the vaccination experience by providing clear information about the benefits and risks of vaccination, addressing concerns and questions, and minimizing pain or discomfort during the vaccination process. So, here, the authors conclude that:

**H_11_**: Vaccination receiver satisfaction strongly and significantly affects intention to receive future vaccination doses for vaccine hesitancy.

**Figure 2:**
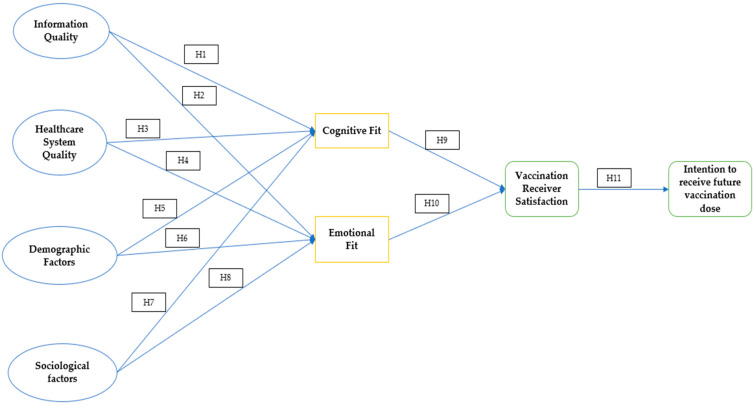
Theoretical Model. Source: Authors’ conception.

Here, the authors proposed the theoretical model as per the below Figure 2.

## 4. Methodology

This section aims to develop self-constructed scales measuring emotional and cognitive fit for vaccine hesitancy. The framework has been instrumental in developing these scales. To create new ones, we refer to the three previous studies to introduce more detailed procedures for developing scales [91,92,93]. We also refer to their concepts for conceptualization and scale development. The initial items were conceptualized by conducting focus groups and gathering information about the study. The other scales were then established through a literature review. The participants were then asked to evaluate the quality of data collected and disseminated within the health care system. They were then asked to provide various details about the information [91]. These included the types of information that they could obtain, their nature, and the factors that affected their emotional and cognitive fit for the vaccine recipients. The submitted items were then evaluated by two expert judges [92]. They agreed that the things represented the construct well. They then graded the research items using a scale of 1 to 5.

### Model, Scale Specification

The survey was conducted on an online survey monkey website. The survey’s link was posted on social media platforms such as Facebook and Twitter. The participants could then respond to the survey by entering the URL provided. The study’s meaning was first introduced to the participants before completing the survey. The survey was conducted to ask about the various benefits of getting vaccinated. The study’s subjects were those who had received at least two vaccine doses. The questionnaire also emphasized that they should have received a message or registered to get the third and fourth doses. The survey’s questions were designed to be answered thoroughly. The study’s results, which were gathered through 1510 samples (Males 47%, 710; Female 53%, 800), did not differ from those of the users who used social media platforms. The most common demographic that participated in the study was individuals aged 20 to 30 (48%, 725). The survey results revealed that 38% of the respondents received a vaccine message at least once a week. 28% said they got a reminder message once every 15 days, while 31% said they got messages once every month, and 3% got messages once in two months. The initial pool of items was developed based on the concept of the study and the qualitative study.

**Figure 3:**
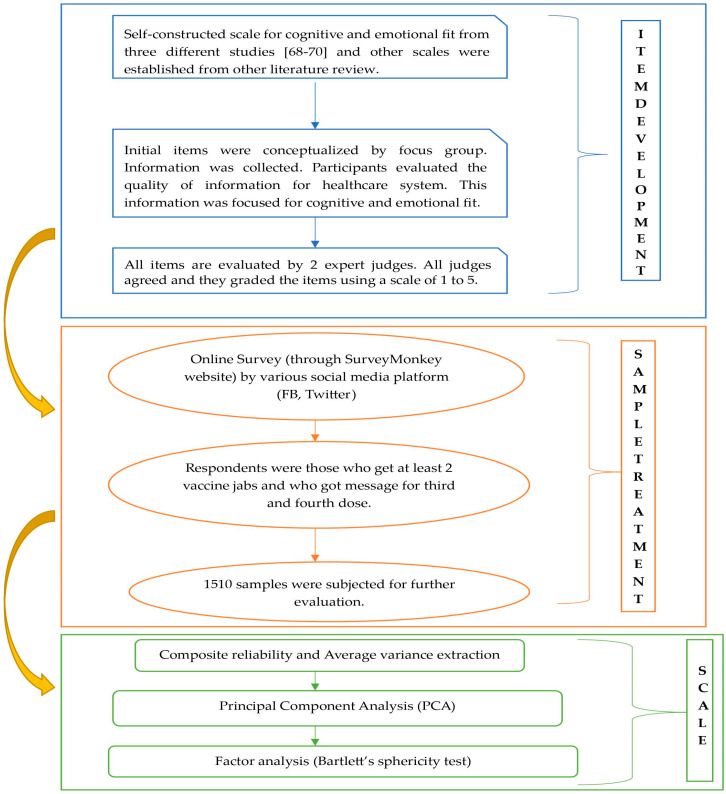
Research Process for This Study. Source: Authors’ conception. Source: Authors’ analysis.

However, before the final dimensions of the study could be calculated, further data analysis was conducted. Scale purification was performed to get a structure of the fit scale. A reliability test and principal component analysis were also performed to understand the scale’s characteristics better. The scale’s reliability was evaluated using Cronbach’s coefficient exceeding 0.7 [93]. A factor analysis was performed using Bartlett’s sphericity test, indicating that the factor loading on each item should be greater than 0.6 [93]. The excluded items were those with cross-loading and ambiguities. The emotional and cognitive fit dimensions were named based on the items’ measurements. Table 1 and Table 2 represent Factor loading for cognitive and emotional fit and constructs’ sources. Figure 3 shows the whole research process for this study.

## 5. Cross-Validation of Scale

The study utilizes cross-validation tools such as IBM SPSS 22 and AMOS 22. The latter was used to analyze the structural and measurement models, as shown in Table 3, while the former was used to provide descriptive statistics on the samples, as shown in Table 4. Furthermore, researchers here evaluated the validity and reliability of the questionnaire and the hypothetical models of the Research.

The goal of the measurement model is to determine the validity and reliability of the data. A study with seven indices was utilized to analyze the fit of the overall framework. The original model’s reliability and fit were low, necessitating the modification of the model. The fit indices of the modified version are shown in Table 5. The reliability of the items and dimensions of the modified model was analyzed. The factor loading and Cronbach’s were then used to measure the reliability of the items and measurements. The factor loadings of variables and indices should be higher than 0.7 [93]. These two factors’ convergence validity was then tested using composite reliability and average variance analyses. The former’s value should be greater than 0.7, while the latter should be over 0.5 [93]. We checked the practical meaning, item, and construct of the low factor loading of SQ4 (0.532). Factors with values greater than 0.5 are generally considered necessary for practical significance. In this study, we examined the functional importance of the factor loading of HSQ4 with a sample size of 1510. After considering the factor loading, we determined that item HSQ4 is of practical significance regarding system quality.

As shown in Table 6, a construct’s discriminant validity differs from other constructs regarding practical significance. This concept should be evaluated using the square root of the various constructs’ average values. The researchers noted a high correlation between the multiple constructs’ average values. They also found that the cross-loading value of the multiple indicators was smaller than that of the constructs.

### Structural Model

**Figure 4:**
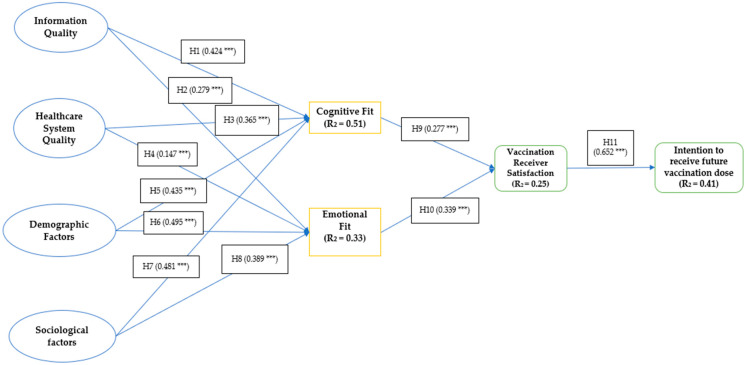
Structural Model. Source: Authors’ analysis. *** *p* < 0.001.

The variance explanation and the path coefficient are presented in the structural model. The study results, summarized in Figure 4 and Table 7, show that the eleven conjectures exhibited positive relationships. The variance explained by the various factors was 33% and 51% for the emotional and cognitive fit of the subjects. For the satisfaction of vaccine receivers, the R^2^ was 25%, while the intention to receive future doses was 41%. Norm development is the final step in establishing the nomological validity of the constructs. It involves validating a well-established relationship between the various and measured constructs. This study aims to confirm the usefulness of the socio-demographic, emotional, and cognitive fit Scale.

## 6. Discussion

The results of this study suggest that the quality of health care systems and information can positively affect individuals’ cognitive and emotional fit. It is consistent with other studies [2,3,4,5]. The main reason for this conclusion is that the quality of information and social media makes people feel more confident about vaccinating. Recently, the importance of vaccines for public health has been highlighted by the COVID-19 pandemic [94,95,96]. However, vaccine hesitancy has also become a growing concern. One way to combat this issue is by providing high-quality health care information services to the public. Access to reliable and accurate information is essential for individuals to make informed decisions about their health [97]. Unfortunately, misinformation and fake news have become increasingly prevalent in the age of social media. It is why health care information services must ensure the provision of trustworthy and high-quality information [8]. When it comes to vaccines, misinformation can have serious consequences. For example, the false belief that vaccines cause autism has decreased vaccination rates in some areas, resulting in outbreaks of preventable diseases.

On the other hand, clear and accurate information about vaccines can improve public trust and increase vaccination rates. Health care information services play a critical role in ensuring the quality of information available to the public [98,99,100]. These services can provide information about the safety and efficacy of vaccines and address common concerns and misconceptions. Additionally, they can help individuals locate vaccination sites and schedule appointments [101].

During the COVID-19 pandemic, health care information services have provided the public with accurate information about the virus and vaccines. It has been crucial in promoting vaccination and reducing vaccine hesitancy [102]. When individuals have access to high-quality health care information services, they are more likely to make informed decisions about their health. It is particularly true for vaccines [12,13,14,15]. Studies have shown that providing accurate vaccine information can increase vaccination rates and reduce vaccine hesitancy.

Moreover, individuals who receive accurate information are more likely to feel confident in their vaccination decision. It can lead to a sense of empowerment and control over one’s health, which can positively affect overall well-being [103,104,105]. In conclusion, health care information services promote public health by providing high-quality vaccine information. By addressing concerns and providing accurate information, these services can increase vaccination rates and reduce vaccine hesitancy [106]. As we continue to navigate the COVID-19 pandemic, prioritizing the quality of health care information available to the public is more critical than ever. Understanding cognitive and emotional fit is crucial in developing effective information-sharing strategies for vaccine takers [107]. By tailoring information to individuals’ preferred methods of receiving information and addressing their emotional concerns, individuals may be more likely to feel comfortable and confident in taking the vaccine. The findings suggest that cognitive and emotional fit and sociodemographic factors affect vaccine information processing in the people who go for vaccine booster dosages [108]. It is important to note that cognitive fit and emotional fit are not one-size-fits-all solutions. Individuals may have unique preferences for how they receive information and what information they need to feel confident in taking the vaccine. As such, it is essential to gather feedback from vaccine takers and tailor information-sharing strategies accordingly [109]. In conclusion, cognitive and emotional fit are crucial factors impacting vaccine hesitancy. By understanding these factors and tailoring the information-sharing approach to meet individuals’ needs, we can work towards increasing vaccine uptake and fighting against the COVID-19 pandemic.

## 7. Academic and Practical Implications

Vaccine hesitancy is an increasing concern for public health officials around the world. Vaccination has become one of the main strategies for controlling the COVID-19 pandemic, so understanding the reasons behind vaccine hesitancy has become a top priority for researchers. Several studies have investigated the factors contributing to vaccine hesitancy, including cultural, social, and psychological factors [110]. These studies have significant academic implications beyond immediate public health concerns.

Developing effective communication strategies is one of the most significant implications of vaccine hesitancy studies. These studies have shown that effective communication is vital in addressing vaccine hesitancy [111]. Researchers have found that communication strategies that are culturally sensitive, transparent, and participatory are more effective in building trust and increasing vaccine acceptance. Such techniques can be developed only by thoroughly understanding the cultural and social factors influencing vaccine hesitancy.

Another significant academic implication of vaccine hesitancy studies is the need to address the root causes of vaccine hesitancy. These studies have identified various factors contributing to vaccine hesitancy, including misinformation, mistrust of the medical establishment, and political polarization. Addressing these root causes requires a multidisciplinary approach involving public health officials, social scientists, communication experts, and policymakers. Vaccine hesitancy studies have significant academic implications beyond immediate public health concerns. These studies highlight the need for effective communication strategies and a multidisciplinary approach to address the root causes of vaccine hesitancy [112]. By working together, researchers from different disciplines can provide a more comprehensive understanding of vaccine hesitancy and develop more effective strategies to promote vaccine acceptance. Here, the contribution of this study is not only for vaccine hesitancy studies but also added a different dimension to information systems and sociodemographic factors.

The COVID-19 pandemic has brought vaccine hesitancy into the limelight, and more people than ever are discussing the importance of vaccines. However, vaccine hesitancy is not new, and researchers have studied its reasons for years. Studies have shown that cognitive, demographic, and emotional factors influence vaccine hesitancy. Here are some practical implications of these studies for addressing vaccine hesitancy: To address these cognitive factors, health care providers and public health officials should:

- Provide accurate and transparent information about vaccines.
- Address misinformation and conspiracy theories clearly and respectfully.
- Build trust with patients by listening to their concerns and answering their questions.

In this study, researchers found that younger people and those with lower levels of education are more likely to be vaccine-hesitant. To address these demographic factors, health care providers and public health officials should:

- Tailor their messaging to different age groups and socioeconomic levels
- Partner with community organizations to reach underserved populations.
- Address cultural beliefs and practices that may contribute to vaccine hesitancy.

Similarly, to address these emotional factors, health care providers and public health officials should:

- Acknowledge and validate people’s fears and concerns.
- Address the emotional impact of the pandemic on people’s mental health.
- Provide support and resources for those experiencing vaccine-related anxiety or distress.

Addressing these cognitive, demographic, and emotional factors, health care providers and public health officials can help address vaccine hesitancy and promote vaccine confidence. However, it is essential to recognize that vaccine hesitancy is complex and multi-faceted, and there is no one-size-fits-all solution. Ongoing Research and collaboration between researchers, health care providers, and public health officials will be critical to addressing vaccine hesitancy and promoting vaccine confidence in the years to come.

The COVID-19 pandemic is a complex and challenging public health crisis. Vaccination is one of the most critical tools we must use to mitigate the spread of the virus. By understanding the factors influencing COVID-19 vaccine intention, we can develop more effective interventions to increase vaccination rates and protect public health. The findings suggest that cognitive and emotional fit is essential when improving COVID-19 vaccine intention. Interventions that can help people manage their anxiety and anger about COVID-19 may increase their motivation to get vaccinated. Additionally, interventions that can help people to understand the benefits of vaccination and to feel more confident in the safety and efficacy of vaccines may also be effective in increasing their intention to get vaccinated. Here are some examples of how cognitive and emotional fit could be applied to increase intention to get vaccinated against COVID-19:

- Use plain language and avoid the jargon of vaccination and health care.
- Provide information that is consistent with people’s existing beliefs and values.
- Use visuals and other multimedia to help people understand the information.
- Provide opportunities for people to ask questions and get clarification.
- Tailor the information to the specific needs and concerns of the target audience.

By taking these steps, we can increase the likelihood that people will understand, remember, and be persuaded by information about the COVID-19 vaccine, which could lead to increased intention to get vaccinated.

Due to recent changes with COVID-19 and the recent WHO declaration, vaccine hesitancy studies are very important for vaccine producers and policymakers. Since the number of new cases and deaths from COVID-19 has been declining in many parts of the world, this is due to several factors, including the widespread availability of vaccines and treatments and the development of natural immunity in the population. The Omicron variant of COVID-19 is now the dominant strain worldwide. Omicron is less severe than previous variants but still highly contagious. It means that despite declining cases, there is still a risk of transmission and painful illness. The WHO has declared that the COVID-19 pandemic is no longer a global health emergency. It means the WHO no longer believes that the virus poses an extraordinary risk to global health. However, the WHO has also warned that the virus is still a threat and that countries should continue to take measures to protect their populations.

The recent changes with COVID-19 and the WHO declaration are positive signs, but it is essential to remember that the virus is still a threat. It is vital to continue to get vaccinated and boosted, to wear a mask in public indoor settings, and to practice good hygiene. By taking these steps, we can help to protect ourselves and our loved ones from COVID-19.

In addition to the above, here are some other things to keep in mind:

- The virus is still evolving, so new SARS-CoV-2 variants that are more transmissible or severe than Omicron could emerge.
- Not everyone has access to vaccines and treatments. It means that the virus will continue circulating in some parts of the world, and new outbreaks are at risk.
- The virus can have long-term effects on people’s health. Even people who have mild cases of COVID-19 can experience long-term symptoms, such as fatigue, shortness of breath, and brain fog.

It is essential to be aware of these risks and to continue to take steps to protect yourself and your loved ones from COVID-19. By doing so, we can help to keep the virus under control and prevent further outbreaks.

## 8. Limitations and Future Scope of Research

The study’s limitations are based on the specific regions and countries where it is conducted, such as India, Italy, Vietnam, and Greece. It is important to note that caution should be exercised when expanding the sample to other people. Future studies may also take representatives from other countries. Although the study focuses on the quality of the vaccine information, other factors, such as sociodemographic factors and health care system quality, may also be considered in future studies.

We considered a specific population for our study. It is because; most of the population around the world and the countries considered; got two doses as per the directives of the federal government. However, after taking two doses, many people start spreading the news on how they feel about taking two doses and are not in support of getting further booster doses, especially young and older ones. Additionally, due to the increasing number of heart attacks and hypertension among the younger population, we considered those who have taken two doses.

## 9. Conclusions

The integration of reality and virtual world information, and the interaction between people and their perceptual information processing techniques are important because these help present the virtual world comprehensively so that users can feel and experience it. This study analyzed the fit of vaccines’ virtual and actual information, especially for COVID-19 and socio-demographical factors, in terms of their stimulus and the player’s emotional and cognitive fit. The factors influencing the user’s emotional and cognitive fit are health care system quality, information quality, and socio-demographical factors.

An emotional bond between players can be formed in uncertain environments, but the main factor influencing this is fit. Since vaccine hesitancy is connected to the real world, people can relate to it regardless of their location. The quality of the information and the virtual news of vaccines on various platforms are also essential factors that influence players’ emotional and cognitive fit. The authenticity and smoothness of the report are also important factors that can affect how people connect with virtual information platforms. The findings of this study can be utilized by developers of vaccine apps, such as those working on booster doses, to improve their creations.

## Figures and Tables

**Table 1:** Factor Loading for Cognitive and Emotional Fit.

Factors	Item	Details	Factor Loading	Cronbach Alpha
Cognitive Fit	CF1	When the virtual platform information matches the real ones, capturing proper information processing	0.783	0.854
	CF2	When the virtual platform information matches the real ones provides assurance	0.851	
	CF3	When the virtual platform information matches the real ones provides trust	0.662	
	CF4	The details of vaccine information are accurate in terms of virtual platform information matches and scientific validation	0.718	
Emotional Fit	EF1	I feel when the virtual information matches the real ones make me happy	0.785	0.814
	EF2	I feel when the virtual information matches the real ones give me emotional stability	0.862	
	EF3	I feel that the virtual information matches the real ones assures my health and safety	0.854	
	EF4	I feel that the virtual information matches the real ones assures my mental health	0.785	

Source: Authors’ analysis.

**Table 2:** Constructs’ Sources.

Constructs	Item	Source
Information Quality	IQ1	[59,61,71,73]
	IQ2	
	IQ3	
	IQ4	
Health Care System Quality	HSQ1	[68,70,73]
	HSQ2	
	HSQ3	
	HSQ4	
Demographic Factors	DF1	[67,68,71,73]
	DF2	
	DF3	
Sociological Factors	SF1	[65,66,69,71]
	SF2	
	SF3	
	SF4	
Vaccination Receiver Satisfaction	VRS1	[67,69,71]
	VRS2	
	VRS3	
	VRS4	
Intention to Receive Future Vaccination Dose	IRFV1	[73,75,77]
	IRFV2	
	IRFV3	
	IRFV4	

**Table 3:** Model Fit Table.

Model Fit Criteria	Measurement Model	Suggested Scope
Chi-square (χ^2^)	2540.012	–
Degree of freedom (d.f.)	2189	–
Standard chi-square test (χ^2^/d.f.)	1.154	≤3
Goodness of fit index (GFI)	0.876	≥0.8
Adjusted goodness of fit index (AGFI)	0.838	≥0.8
Comparative fit index (CFI)	0.948	≥0.9
Normed fit index (NFI)	0.912	≥0.9
Incremental fit index (IFI)	0.953	≥0.9
The root-mean-square error of approximation (RMSEA)	0.071	≤0.08

Source: Authors’ analysis.

**Table 4:** Descriptive Statistics.

Item	Item for Measurement	Number of Respondents	Percentage (%)
Gender	Male	710	47
	Female	800	53
Age	18 to <20 years old	181	12
	20 to <30 years old	725	48
	30 to <40 years old	211	14
	40 years old and above	393	26
Reminder message (Time)	At least once a week, receive the notification for vaccination reminder	574	38
	A reminder message once every 15 days	423	28
	Once every month	468	31
	Once in two months	45	3

Source: Authors’ analysis.

**Table 5:** Reliability and Validity of Dimension Items.

Dimension	Item	Factor Loading	Cronbach’s α	CR	AVE
Information quality (I.Q.)	IQ1	0.754	0.757	0.757	0.51
	IQ2	0.718			
	IQ3	0.723			
	IQ4	0.668			
Health care system quality (HSQ)	HSQ1	0.781	0.74	0.758	0.519
	HSQ2	0.822			
	HSQ3	0.775			
	HSQ4	0.532			
Demographic factors (D.F.)	DF1	0.753	0.791	0.778	0.547
	DF2	0.813			
	DF3	0.769			
	DF4	0.694			
Sociological factor (S.F.)	SF1	0.861	0.813	0.789	0.618
	SF2	0.748			
	SF3	0.762			
	SF4	0.798			
Cognitive fit (C.F.)	CF1	0.878	0.833	0.839	0.635
	CF2	0.752			
	CF3	0.761			
	CF4	0.754			
Emotional fit (E.F.)	EF1	0.898	0.93	0.936	0.83
	EF2	0.957			
	EF3	0.877			
	EF4	0.868			
Vaccine receiver satisfaction (VRS)	VRS1	0.877	0.932	0.936	0.83
	VRS2	0.881			
	VRS3	0.96			
	VRS4	0.894			
Intention to receive future vaccination dose (IRFV)	IRFV1	0.968	0.904	0.91	0.774
	IRFV2	0.933			
	IRFV3	0.736			
	IRFV4	0.718			

Source: Authors’ analysis.

**Table 6:** Discriminant Validity.

	IRFV	VRS	EF	CF	DF	SF	IQ	HSQ
IRFV	0.88							
VRS	0.669	0.894						
EF	0.318	0.517	0.911					
CF	0.329	0.478	0.659	0.797				
DF	0.518	0.616	0.55	0.534	0.74			
SF	0.528	0.624	0.567	0.512	0.512	0.712		
IQ	0.325	0.488	0.45	0.634	0.421	0.418	0.714	
HSQ	0.256	0.362	0.357	0.584	0.302	0.301	0.711	0.72

Source: Authors’ analysis.

**Table 7:** Model Fit for Different Constructs.

	IQ	HSQ	DF	SF	CF	EF	VRS	IRFV
IQ1	0.754	0.536	0.318	0.434	0.478	0.339	0.368	0.245
IQ2	0.718	0.511	0.303	0.371	0.456	0.323	0.351	0.234
IQ3	0.719	0.519	0.412	0.631	0.461	0.322	0.312	0.212
IQ4	0.668	0.475	0.282	0.453	0.424	0.301	0.326	0.217
SQ1	0.567	0.814	0.311	0.281	0.318	0.317	0.329	0.218
SQ2	0.585	0.822	0.249	0.192	0.48	0.294	0.298	0.21
SQ3	0.551	0.775	0.234	0.464	0.453	0.277	0.281	0.198
SQ4	0.377	0.531	0.16	0.501	0.31	0.19	0.192	0.136
DF1	0.317	0.228	0.753	0.427	0.402	0.414	0.464	0.39
DF2	0.343	0.246	0.813	0.494	0.434	0.447	0.501	0.421
DF3	0.292	0.21	0.694	0.281	0.371	0.382	0.427	0.36
DF4	0.431	0.342	0.526	0.192	0.631	0.957	0.494	0.304
SF1	0.551	0.775	0.234	0.377	0.453	0.277	0.281	0.198
SF2	0.377	0.531	0.16	0.478	0.31	0.19	0.192	0.136
SF3	0.478	0.441	0.403	0.477	0.754	0.497	0.361	0.248
SF4	0.477	0.439	0.402	0.557	0.752	0.496	0.36	0.247
CF1	0.557	0.513	0.469	0.477	0.878	0.579	0.42	0.289
CF2	0.477	0.439	0.402	0.481	0.752	0.496	0.36	0.247
CF3	0.481	0.481	0.404	0.578	0.734	0.487	0.41	0.251
CF4	0.478	0.441	0.403	0.563	0.754	0.497	0.361	0.248
EF1	0.404	0.321	0.494	0.898	0.592	0.898	0.464	0.285
EF2	0.431	0.342	0.526	0.957	0.631	0.957	0.494	0.304
EF3	0.395	0.313	0.482	0.877	0.578	0.877	0.453	0.278
EF4	0.387	0.321	0.475	0.871	0.563	0.871	0.483	0.281
VRS1	0.428	0.318	0.54	0.459	0.42	0.453	0.877	0.587
VRS2	0.436	0.324	0.551	0.428	0.428	0.462	0.894	0.598
VRS3	0.469	0.348	0.591	0.318	0.459	0.496	0.96	0.642
VRS4	0.436	0.324	0.551	0.251	0.428	0.462	0.894	0.598
IFRV1	0.315	0.247	0.501	0.248	0.318	0.307	0.648	0.968
IFRV2	0.303	0.239	0.483	0.285	0.307	0.296	0.624	0.933
IFRV3	0.233	0.183	0.372	0.304	0.236	0.228	0.48	0.718
IFRV4	0.213	0.191	0.365	0.598	0.241	0.229	0.412	0.727

Source: Authors’ analysis.

## Data Availability

Data sharing not applicable due to privacy restrictions.
